# Effects of hydrological regime on development of *Carex* wet meadows in East Dongting Lake, a Ramsar Wetland for wintering waterbirds

**DOI:** 10.1038/srep41761

**Published:** 2017-02-06

**Authors:** Lei Jing, Cai Lu, Yan Xia, Linlu Shi, Aojie Zuo, Jialing Lei, Hong Zhang, Guangchun Lei, Li Wen

**Affiliations:** 1 School of Nature Conservation, Beijing Forestry University, Beijing, China; 2 College of Forestry, Central South University of Forestry and Technology, Changsha, China; 3 East Dongting Lake National Nature Reserve Authority, Yueyang, China; 4 Science Division, Office of Environment and Heritage, Sydney, New South Wales, Australia

## Abstract

Wet meadows are one of the most important ecological components in floodplain, and are among the most dynamic ecosystems. Understanding the development of wet meadows and contributing environmental factors can provide better support for wetland management. *Carex* meadows in East Dongting Lake National Nature Reserve (EDLNNR) provide vital wintering ground for thousands of migratory waterbirds, and their ecological functions are under threated due to hydrological alternation. We measured wet meadow expansion in EDLNNR from 1989 to 2014, and explored its responses to hydrological and climatic factors within the generalised additive models (GAM) framework. We found an overall expansion of wet meadows over the study period. However, in contrast to many previous studies, our results showed that water level fluctuations at the hydrologic indicator site had only limited impacts on their development. Instead, sampling year, timing of water level recession, and local rainfall exerted significant effects. The effects of sampling year reflected the changes in sedimentation within Dongting Lake; and effects of timing of water withdrawal might be explained by the life history of the dominant sedge species. Our study suggested that the impacts of large scale hydrological alternation on vegetation may operate indirectly through its effects on sediment balance.

Wet meadows are one of the most important ecological components in floodplain systems for their biological diversity^1^ and the ecosystem services they provide, including nutrient cycling, pollution removal, and carbon sequestration^2^. However, these values have been degraded or destroyed in recent decades, mainly due to land use intensification^3^ and hydrological alternation^4^. For example, floodplain meadows belong to the most threatened plant communities in Europe^5^. In the central Yangtze River, wet meadows are identified as the most important habitat for wintering goose (*Anser* of the Anatidae family)^6^,^7^,^8^. Recent studies suggest that the annual colonization and development of wet meadows is impacted by alternated hydrological regime, presumably resulted from the construction of the Three Gorges Dam (TGD)^7^. And, the changes in wet meadows development might be an important factor contributing to the population declines in Anatidae species^9^.

Floodplain wet meadows are a dynamic and heterogeneous ecosystem^10^, often composed of a complex mosaic of plant communities^5^,^11^. Previous studies demonstrated the decisive role of water regime on the colonization and development stage of floodplain meadows^6^,^12^,^13^. Water regime within a floodplain varies temporally and spatially across the floodplain according to its geomorphology^14^. Flooding usually causes erosion and sedimentation^15^, temporarily anaerobic soil conditions^16^, and affects the nutrient regime of the soils^17^. Flooding supports plant species which are adapted to it^18^. Research on wet meadows dynamics in wetland has been of interest for decades; and hydrological regime, which mainly consists of water-level fluctuation, inundation, and hydro-period related variables (start time, duration, and end time of flood)^19^, is considered as one of the most influential factors^20^,^21^,^22^,^23^.

Dongting Lake located in the downstream of the TGD on Yangtze River, is the second largest freshwater lake in China, and is also one of the only two large lakes (i.e. Dongting and Poyang) directly connected with the Yangtze River. The Dongting Lake plays a vital role in flood controlling and climate regulation in the middle and lower reach of Yangtze River basin due to its large surface are and storage capacity^24^. It also provides numerous habitats for floras and faunas at local, regional, and global scales as it encompasses various wetland types, such as wet meadows, shadow waters, and mudflats^6^. The East Dongting Lake (EDL, a main part of Dongting Lake, situated on the south bank of Yangtze River, Fig. 1), is an important wintering site for migratory waterbirds. Tens of thousands of waterbirds wintering at EDL every year, of which 27.8–56.3% choose wet meadows as their foraging habitat^6^. In particular, more than 90% of the East Asian population of the threatened Lesser White-fronted Goose (*Anser erythropus*) overwinter at the EDL, and foraging extensively on wet meadows^8^.

Without human intervention, functional floodplains in the mid-lower Yangtze region would be dominated by wet meadows with a characteristic zonation of plant species from lower to higher elevated area. However, in recent years, expansion of *Carex* meadows with the expense of other habitat types are found in Dongting Lake, and water level change due to the TGD is considered as the main influential factor^25^,^26^. However, these results are based on monthly or even seasonal water level change; and there is still lack of analysis on long-term inter-annual water level variability. In addition, the exchange of water and sedimentation between Dongting Lake and upper rivers, as well as the impacts of local climate variability, should be considered for a comprehensive study.

The objective of this study is to determine the main environmental factors contributing to the expansion of wet meadows in EDLNNR during the last three decades. We first measured the areas of wet meadows in EDLNNR during the low water winter season from 1989 to 2014 using Satellite imagery. The inter-annual variation in wet meadows areas was then modelled within the generalized additive modelling (GAM) framework to investigate the relative importance of hydrological and climatic factors.

## Results

### There was no persistent trend in water level time series

There was a clear intra-annual dry-wet cycle in the monthly water level time series (Fig. 2). However, there was no obvious abrupt changes and inter-annual trend (Fig. 2). The nonparametric correlated seasonal Mann-Kendall test confirmed that there was not overall trend in the monthly time series (p = 0.249). However, water level at Chenglinji for October showed a significant decreasing trend (S = −155, *p* = 0.001) according to the seasonal Mann-Kendall test (Table 1).

### Spatio-temporal variability of land cover in EDLNNR

During the study period, the total area of wet meadows, reed marshes and mudflats increased greatly in EDLNNR (4,765 ha, 3,840 ha and 1,365 ha for wet meadow, reed marshes and mudflat, respectively) with the expense of open water (reduced 9,971 ha in 24 years). While the increase in reed marshes was spatially concentrated, the expansion of wet meadows was more widespread and extensive (Appendix 1). The wet meadow expansion occurred at the edge of waters all over EDLNNR except the river channels. It was worth to point out that the spatially-concentrated increase of reed marshes was due to artificial plantation on wet meadow, and this kind of land reclamation practice was common in the Dongting Lake region before the early 2000.

### The main contributors included time, date of water level recession and local rainfall

The final model included three significant predictors: Years, Rainfall and Timing1 (Jualian day when water level at Chenglingji drops to 25 m), and it had relatively good performance (76.2% explained deviance, Table 2).

The general increasing trend during the study period was reflected in the modelled positive relationship between the area of wet measure and sampling year (coefficient = 0.0074, Table 2). The effect of Timing1 was also positive but rainfall in the winter had a significant negative impact (Table 2). The effects of Years and Timing1 showed strong nonlinearity while that of the Rainfall was linear (Fig. 3). The model revealed a turning point for Years at around 14 (i.e. year 2002) when the positive relationship became flatten. For Timing1, i.e. the date when water level at Chenglingji declined to 25 m, a clear turning point at Julian day 275 (i.e. first of October) could be identified (Fig. 3).

The 3-D response surfaces of the modelled relationships were presented in Fig. 4. With Rainfall kept constant at the long-term median of 97.55 mm, the increasing of the wet meadows with time stopped midway at 14 (i.e. 2002), before which the wet meadows increased about 550 ha per year (Fig. 4A). For the timing of water level recession, the GAM indicated that the wet meadows size did not change with the timing before around mid-October, after which it increased rapidly with Julian day (about 160 ha/day, Fig. 4A). With Timing1 kept at its median value of 299 (i.e. 24 October), the effect of Years on wet meadows was positive (about 510 ha per year) till 2006, after that the positive effect diminished (Fig. 4B). The effect of winter rainfall was negative and relatively constant at about −0.6 ha/mm (i.e. when winter rainfall increases 10 mm, the size of wet meadows decreases 6 ha, Fig. 4B).

## Discussion

### Water level at Chenglingji might not be the main reason responsible for the recent expansion of wet meadows in EDLNNR

In our study, a general increasing trend was found in wet meadows distribution in EDTNNR during the study period. This result is consistent with the findings from previous researches^25^,^26^. Consonantly, these previous studies considered that the decreased water level due to the operation of the Three Gorges Dam was the main factor contributing to the expansion of wet meadows. In our analysis, however, annual minimum water level at the hydrological indicator site Chenglingji was not selected in the final model suggesting the limited effect of this variable. This disagreement might be due to the different hydrological metrics investigated. The non-parametric Mann-Kendall test indicated that there was no constant trend in the monthly water level data (p = 0.249). However, the seasonal Mann - Kendall test showed that the decreasing trend was significant in October (Table 1). Importantly, the average temperature in October is 18.6 °C (mean for 1961–2015, Yueyang), ideal for *Carex spp* (the dominant grass species in EDTNNR) to rapidly colonize the exposed lake bed^6^. The coincidence of optimal temperature and large suitable area available resulted from water level drawdown may facilitate the rapid colonization of *Carex*. Therefore, the expansion of wet meadows over the study years might be related to the month – specified water level decrease (i.e. in October); and this was confirmed by further analysis – the Pearson correlation test revealed that there was a weak negative relationship between the size of wet meadows and water level in October (r = −0.43, p = 0.099, n = 16). On contrast, the relationship between wet meadows and annual minimum water level was insignificant (r = 0.07, p = 0.803, n = 16). Nevertheless, when we replaced annual minimum water level with water level in October, results similar to Table 2 were obtained indicating the relative insignificance of water level at Chenglingji on wet meadows development comparing to other variables including Year, Timing and Rainfall.

**Figure 1:**
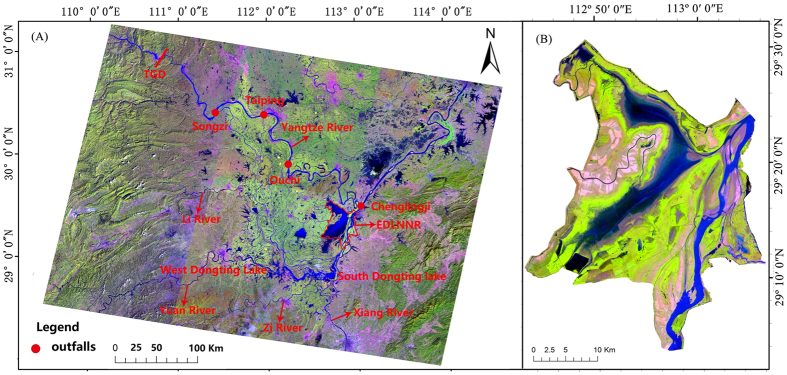
Maps of Dongting Lake. (**A**) Shows the relationship between Dongting Lake and upper rivers, as well as the outfalls, and (**B**) is the map of EDLNNR in winter, the light green parts are the wet meadows, and the blue parts are the river channels and sub-lakes. Maps were created using ArcGIS (v 10.2, Esri, Redlands, CA, USA).

**Figure 2:**
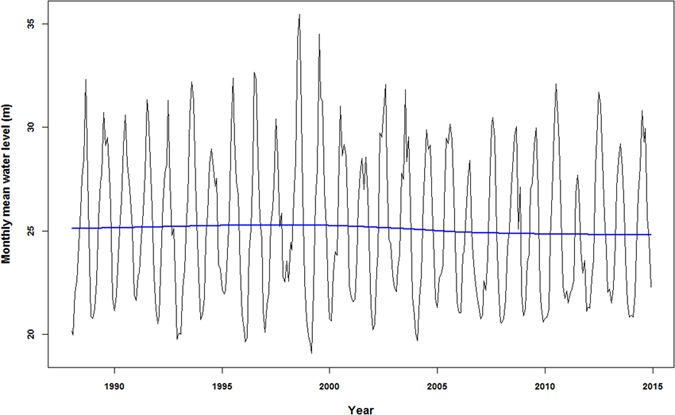
Monthly water level fluctuation at Chenglingji. The blue line is the *LOWESS* smoothed water level, showing no trend and abrupt changes for the study period (1988–2014).

**Figure 3:**
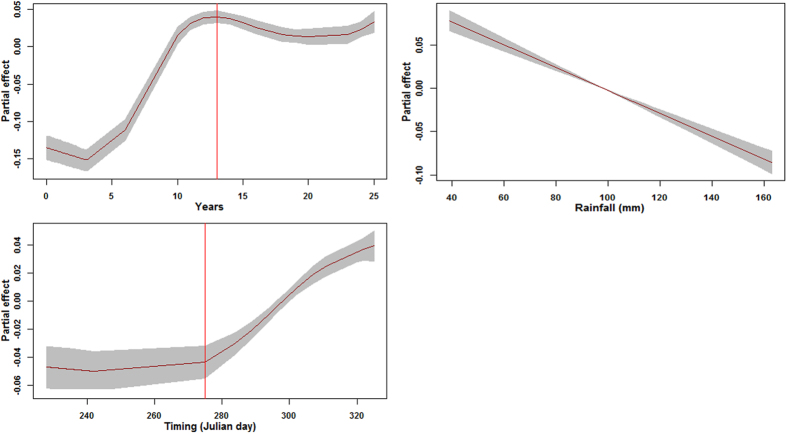
Partial effects of Year, Rainfall and Timing1 on the size of wet meadows in East Dongting Lake. The red curves are the mean and the grey shaded areas are standard errors. The vertical lines indicate the turning points, at which the relationship between the size of wet meadows and the predictor changed rapidly.

The weak relationship between the area of meadows and minimum water level at Chenglingji may reflect the complicated hydrology at sub-lake level^6^. When water level withdraws in dry season, numerous sub-lakes emerge (Fig. 1). These sub-lakes have a range of hydrological connectivity with the main river channel. Many sub-lakes become hydrologically de-coupled with the river when water level at Chenglinji decreases below a certain level; and for a period of months, water level fluctuations in these lakes are controlled by local rainfall and evaporation, which was indicated by the significant relationship between winter rainfall and wet meadows size (Table 2 and Fig. 3).

### The importance of sampling year on wet meadows size – likely associated with sediment inputs to Dongting

The modelling clearly indicated the nonlinear effects of sampling year on the size of wet meadows in EDTNNR; and a turning point (i.e. 2002, Fig. 3) was identified. The areas of wet meadows continuously expanded till 2002, from when the sampling year had limited effect (i.e. the response curve becomes flat). This is the first time to report the turning point, which might be induced by the operation of TGD. The turning point of 2002 is very close to 2003, when the TGD reservoir started to fill. Before 2002, Dongting Lake received large amount of sediment from the “three outfalls” of Yangtze River (Fig. 1); and was a net sediment sink^27^. With sediments deposited in the lake, more and more areas became suitable habitat for sedge species resulting an almost linear increase of wet meadows (Fig. 3). However, after the closure of TGD, concentration of suspended solid particles in river flow of the downstream Yangtze decreased substantially^28^,^29^,^30^. Larger dams have greater sediment trapping efficiency, for example, for reservoir with capacity greater than 10^7^ m^3^, the sediment trapping efficiency is commonly greater than 99%^31^,^32^. For the Yangtze, research found that ~85% of the sediment from upstream was trapped by TGD in recent years^33^. With suspended solid input decreasing, erosion of river and lake banks gradually takes place^34^ resulting in less suitable area for wet meadows development. The modelled partial effects of sampling year on the size of wet meadows in EDTLNNR captured this process (Fig. 3), reflecting the variability of total suspended solids dynamics caused by the TGD.

### The effects of the timing of water level recessing – the importance of temperature for *Carex* germination

**Figure 4:**
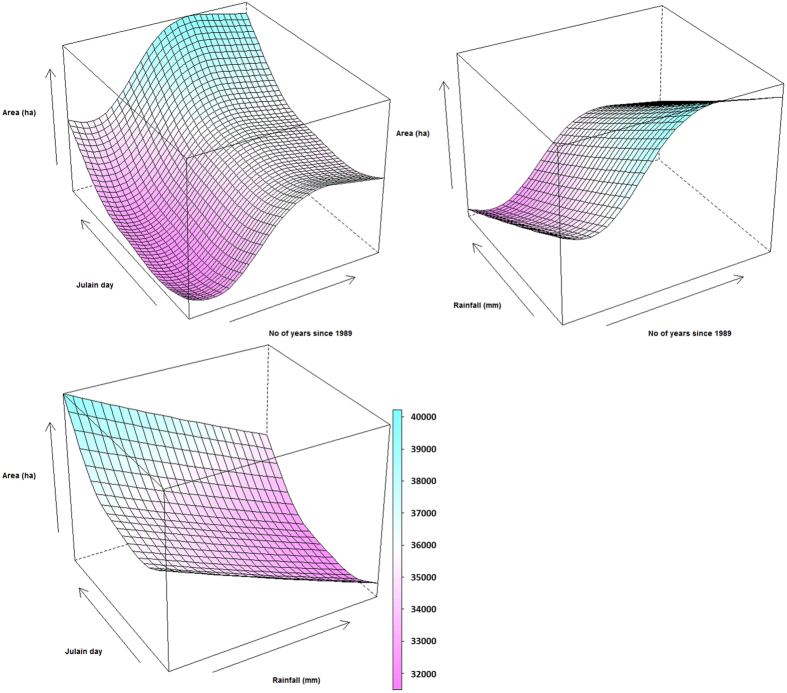
The response surfaces of wet meadows size to predictor variables, (**A**) Years and Timg1, (**B**) Years and Rainfall, and (**C**) Rainfall and Timing1. The response surfaces were constructed using predictions of the final model (Table 2) by letting the two corresponding variables varying gradually within their changing ranges while keeping the third variable at its median value (Table 4).

Our result showed that the timing of water level withdrawal had a significant effect on wet meadow colonization (Figs 3 and 4). The partial effect curve of timing indicated that the effect was strongly nonlinear with a turning point in October 1 (i.e. DOY 275), before when the timing of water level withdrawal had little impacts; and a rapid increasing of wet meadows with late water level withdrawal was observed. This result was seemly contradictory to previous assumption that early water withdrawal would be favourable to the development of wet meadows^7^. This might be related to the life history of *Carex brevicuspis*, the dominate sedge species in Dongting Lake. *Carex brevicuspis* is a perennial rhizomatous sedge found in eastern mainland China and Taiwan^35^. In Dongting Lake, *Carex* grassland is completely inundated and the aboveground shoots senesce during the high water summer season^36^. Regeneration from ramets in Autumn after water level withdrawal is the predominant reproductive form^37^. Previous studies observed the re-germination and spreading of *Carex* vegetation from the senescent ramets after the lake bed has been exposed^7^,^38^,^39^. Most of these studies focused on the impacts of hydrological regimes, and the effects of temperature on the recruitment of *C. brevicuspis* was largely unexplored. The temperature requirements for dormancy break and seed germination vary greatly among wetland *Carex* species^40^. Kettenring and Galatowitsch^40^ reported the optimal temperature requirements for seed germination ranged from was 15–27 °C for 14 temperate *Carex* species. However, there is no information on the temperature requirements of vegetative regeneration of *C. brevicuspis*. Our results, which showed that the early water level recession (therefore the exposure of suitable habitat) had limited effect on *Carex* meadow development, suggested high temperature in late September and early October in the study region might prohibit the vegetative reproduction of this species. Nevertheless, the increase of the size of wet meadows with Day of Year (DOY) after October was consistent with the findings of a recent study, in that all the early emerging plants (sprouted in October) withered, but the later emerging ones (sprouted in November and December) survived in both mature and juvenile populations^39^.

In this study, we explored the long-term dynamics of wet meadows in East Dongting Lake and its response to hydrological, climatic and sedimentary variations. Although the significance of hydrological regimes on wetland vegetation has been well documented^2^,^6^,^38^, our study demonstrated that measurements from a single “indictor” hydrological station was inadequate to explain the temporal variability of vegetation distribution in the floodplain lake complex, maybe due to the de-coupled river-lake relationship during low water seasons. On contrast, our results indicated that seasonal local rainfall and sedimentation played decisive roles in vegetation recruitment and development. The impacts of large scale hydrological alternation such as the operation of Three Gorges Dam on vegetation may operate indirectly through its effects on sediment balance.

## Methods

### Study sites

We chose the East Dongting Lake National Natural Reserve (EDLNNR, 92,064 ha) as study site, because it has the largest wet meadows in the Dongting Lake. Moreover, not like other regions, there was few reed and poplar plantations in the core areas in the past decades, therefore, less complication for satellite image interpretation for more accurate wet meadows estimation. Thus, the development of wet meadows in the reserve can be rationally assumed to be the natural results of hydro-geomorphological changes.

EDLNNR (E112°43′ to E113°14′and N29°00′ to N29°38′, Fig. 1) was designated as a Ramsar Site of International Importance in 1992^41^. It belongs to the subtropical monsoon climate zone, and has clear wet and dry seasons. The Lake receives flows from the Yangtze River via “three outfalls” (Songzi, Taiping and Ouchi, Fig. 1)^42^, as well as the four main tributaries. The Lake discharges back to the Yangtze River via Chenglingji (Fig. 1).

### Remote sensing imagery processing

Remote sensing technology is a common but effective method for monitoring land cover changes in wetlands^43^,^44^,^45^. We selected 16 high quality (i.e. cloud cover less than 5%) Landsat satellite images (Table 3) of EDLNNR in the dry season (download from USGS Global Visualization Viewer, http://glovis.usgs.gov/) to investigate the expansion of wet meadows. We selected the images for these dates so that the water level at Chenglingji hydrological station was less than 22.01 m and close to the corresponding annual minimum water level. In addition, the water level differences at these dates were less than 2 m to ensure the inter-annual comparability. More importantly, as *Carex* regeneration generally stops before middle November^7^, and the size of wet meadow reached its peak and remained relatively stable around these dates. To support our decision of using only one image per year, we compared two land cover maps in one winter (12/06/2013 and 01/23/2014), and the difference for wet meadow, reed march, mudflat and water area is −0.86%, −0.23%, −4.64% and 6.65%, respectively. This result suggests that intra-annual variability of the land cover during the low water winter season is negligible for our focal classes.

In the study, we used Supervised Classification and Visual Revision methods to classify the land cover of EDLNNR into four types, i.e. water body, mudflat, wet meadows, and reed marsh). We verified the classification of 2013-2014 land cover map using 131 randomly selected ground-truth points. The overall accuracy was 83.97% and the Kappa coefficient was 0.75 indicating satisfactory classification^26^. The confusion matrix of the remote sensing classification was presented in Appendix 2.

### Hydrological regime and climatic variables

The hydrological regime in East Dongting Lake can be well represented by the river flow gauged at Chenglingji Hydro-Station^26^,^46^, which services as the hydrologic “indicator” site for studying key ecosystem functions. Prior to further analysis, we used the correlated seasonal Mann-Kendall test (CSMK)^47^ to check if there is any significant trend in the water level time series (1988–2014). A consistent decreasing trend in the lake water level might induce the increase of wet meadows area^48^. We used the CSMK test based on two considerations: firstly, the water level didn’t satisfy the normality for all months (e.g. p = 0.04 for March, Shapiro-Wilk normality test); secondly, there was clear serial correlations among the monthly water levels indicated by the autocorrelation and partial autocorrelation functions (Appendix 3).

Using the daily water level time series at Chenglingji, we derived five variables which have great effects on *Carex* wet meadows colonization and development (Table 4): Timing1 and Timing 2, the Julian days when water level at Chenglingji declines to 25 m and 22 m, respectively; Duration, the number of days for water level falling from 25 m to 22 m; and the minimum winter water level (MinWL). Results in previous study suggest that, when water level at Chenglingji falls below 25 m, the rate of lake bed exposure (thus the potential site for *Carex* development) accelerates^6^. Moreover, when water level drops to 22 m, the area of exposed lake bed in East Dongting Lake reaches its peak and becomes stable^26^.

Generally, local rainfall has considerable contribution to the lake water level, especially during dry seasons^49^. We calculated the total rainfall for the period of November to January using daily climatic data downloaded for Yueyang Climate Station (113°05′E, 29°23′N), and incorporated into the analysis (Table 4).

### Modelling

We built generalised additive models (GAM)^50^ to explore the relationships between the size of wet meadows (i.e. dependent variable) and candidate hydrological and climatic variables (i.e. independent variables). GAMs are an extension of generalized linear models (GLMs)^51^ that a priori does not required to assume a particular shape for the response variable, thus, they are more meaningful to reveal environmental gradients. The initial inspection of the data set revealed that there is a general increasing trend in the area of wet meadows. To capture this trend, we included a variable “Years”, which is the number of years since 1989 (for example, 2013 corresponds to Years = 13), in the model framework.

The general GAM is given by:


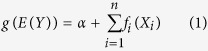


where g (.) is a link function; *E(Y)* is the expected value of response variable *Y. X*_*1*_*, …. X*_*n*_is a set of predictor variables. *f*_*i*_(.) are unspecified smoothing functions, which are estimated in a parametric or non-parametric fashion, and a is a constant, also interpreted as the modelled mean for the responsible variable Y.

To specify a proper link function, we fitted four null GAMs (i.e. a model with only an inception term) with Gaussian (link = “identity”), Gamma (link = “inverse”), inverse Gaussian (link = “1/μ^2^”), and Gumbel (link = “identity”) using the R package “*gamlss*”^52^ and selected the one with the lowest GAIC (Generalized Akaike Information Criterion, the smaller the value, the better the fitting)^53^. The testing results showed that the Gamma distribution was the best option; therefore, Gamma distribution with inverse link function was specified for the modelling.

The predictor variables were selected using a stepwise forward selection procedure: starting from a null models, the smoothing term (we used cubic spine smoothing in this study) of each of the candidate variables were introduced to the model frame one by one, and the model with the lowest GAIC was selected and reported. We also evaluated the performance of the GAMs based on D^2^ values, which represents the percentage of deviance explained and is analogous to the R^2^ as produced by simple linear regression^54^. In addition, we evaluated the significance of each predictor variables using the Wald like tests, conditional on the smoothing parameter estimates^55^. All analysis was done in R (version 3.1.1)^56^.

## Additional Information

**How to cite this article:** Jing, L. *et al*. Effects of hydrological regime on development of *Carex* wet meadows in East Dongting Lake, a Ramsar Wetland for wintering waterbirds. *Sci. Rep.* **7**, 41761; doi: 10.1038/srep41761 (2017).

**Publisher's note:** Springer Nature remains neutral with regard to jurisdictional claims in published maps and institutional affiliations.

## Supplementary Material

Supplementary Information

## Figures and Tables

**Table 1:** Seasonal Mann-Kendall test statistics for time series of monthly water level at Chenglingji.

	S	τ	*p*
Jan	69	0.197	0.150
Feb	11	0.031	0.819
Mar	−35	−0.100	0.466
Apr	−65	−0.185	0.175
May	33	0.094	0.491
Jun	19	0.054	0.692
Jul	−45	−0.128	0.348
Aug	1	0.003	0.983
Sep	−27	−0.077	0.574
**Oct**	**−155**	**−0.442**	**0.001**
Nov	−65	−0.185	0.175
Dec	−13	−0.037	0.786
Overall	−272	−0.065	0.102

**Table 2:** Summary of the final wet meadows GAM.

Predictor	Estimate	Std.	p value
(Intercept)	10.29	0.0917	<0.001
cs (Years)	0.0074	0.0004	<0.001
cs (Rainfall)	−0.0001	0.0000	<0.001
cs (Timing1)	0.0012	0.0001	<0.001
GAIC	279.25		
Deviance explained (D^2^)	76.2%		

Notes: cs = cubic smoothing; GAIC = Generalized Akaike Information Criterion.

**Table 3:** Information of the Landsat images.

No.	Satellite type	Path	Date	Water level
1	TM4-5	123-40	1989.1.26	21.44
2	TM4-5	123-40	1992.1.27	20.08
3	TM4-5	123-40	1995.12.5	21.49
4	TM4-5	123-40	1999.12.24	21.25
5	ETM-7-SCL ON	123-40	2000.2.26	21.06
6	ETM-7-SCL ON	123-40	2001.1.11	21.88
7	TM4-5	123-40	2001.12.21	21.54
8	ETM-7-SCL ON	123-40	2003.1.17	22.00
9	TM4-5	123-40	2003.12.27	20.52
10	TM4-5	123-40	2004.12.13	21.96
11	TM4-5	123-40	2006.12.19	21.07
12	TM4-5	123-40	2007.2.5	20.57
13	TM4-5	123-40	2008.3.11	20.66
14	TM4-5	123-40	2011.1.15	22.10
15	8-OLI-TIRS	123-40	2013.12.06	20.61
16	8-OLI-TIRS	123-40	2014.1.23	21.10

**Table 4:** Candidate predictors for the size of wet meadows in East Dongting Lake.

Variable	Median (range)	Description
Sampling Years	0–25	No of years since 1989
Timing1	299 (228–311)	Jualian day when water level at Chenglingji drops to 25
Timing2	334 (277–364)	Jualian day when water level at Chenglingji drops to 22
Duration	34.5 (9–92)	No of days for water level at Chenglingji withdaws from 25 to 22
Rainfall (mm)	97.5 (39.2–163.0)	Total rainfall (Nov -Jan)
MinWL (m)	20.3 (18.9–21.3)	Minimun water level at Chenglingji
