# Supplementary material for: Effects of hydrological regime on development of Carex wet meadows in East Dongting Lake, a Ramsar Wetland for wintering waterbirds
Source: Sci Rep. 2017 Feb 6;7:41761. doi: 10.1038/srep41761 (PMC5292947; doi:10.1038/srep41761)

**Supplementary materials**

**Effects of hydrological regime on development of *Carex* wet meadows in East Dongting Lake, a Ramsar Wetland for wintering waterbirds**

Lei Jing ^1,2†^, Cai Lu ^1†^, Yan Xia ^2^, Linlu Shi ^1^, Aojie Zuo ^1^, Jialing Lei ^1^, Hong Zhang ^3^,

Guangchun Lei ^1*^, Li Wen ^4*^

*^1^ School of Nature Conservation, Beijing Forestry University, Beijing, China*

*^2^ College of Forestry, Central South University of Forestry and Technology, Changsha, China*

*^3^ East Dongting Lake National Nature Reserve Authority, Yueyang, China*

*^4^ Science Division, Office of Environment and Heritage, Sydney, New South Wales, Australia*

*^*^ Corresponding author. guangchun8099@gmail.com (Guangchun Lei).*

*^*^ Corresponding author. li.wen@environment.nsw.gov.au (Li Wen).*

*^†^These authors contributed equally to this work.*

**Appendix 1 Map shows the change of main land cover types in East Dongting National Nature Reserve between 1989 and 2014. The background is the Landsite image obtained at Jan 17, 2014. Map was produced with ArcGIS (v 10.2, Esri, Redlands, CA, USA).**


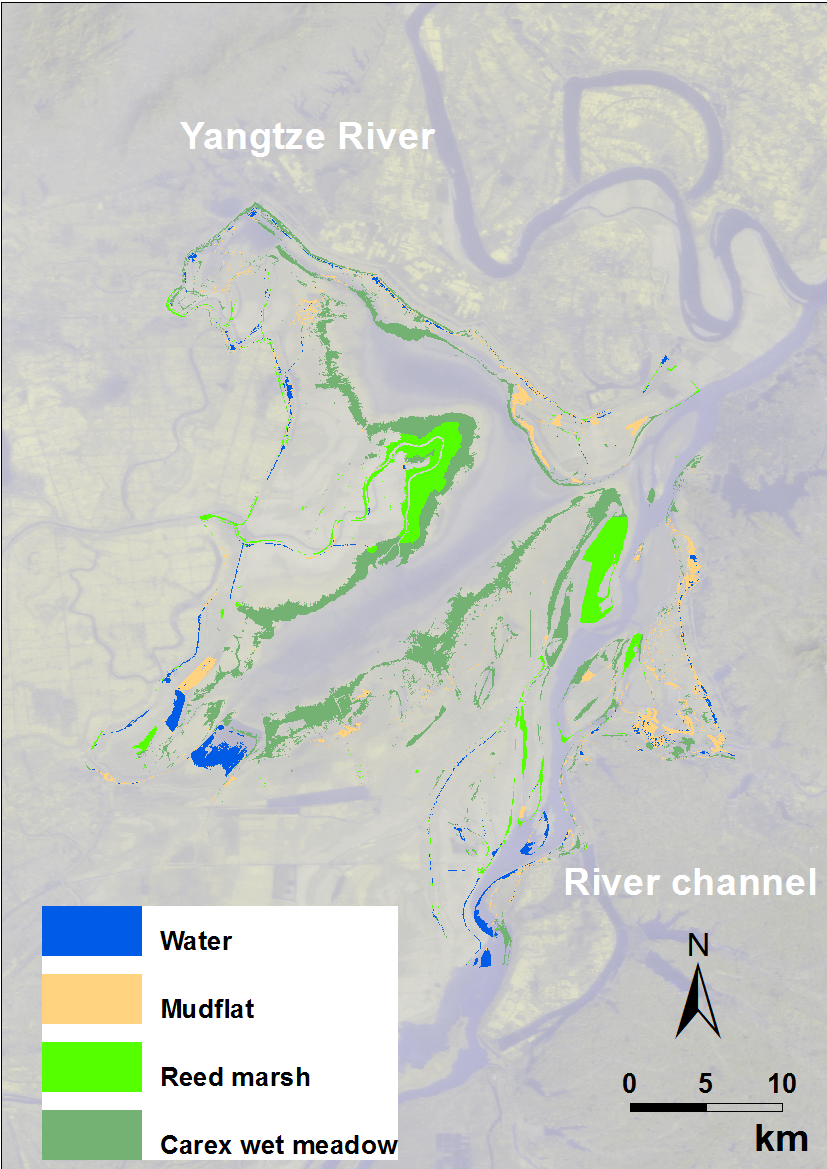


**Appendix 2 Confusion matrix between the vegetation formation classification derived from the Landsite image acquired on Dec 6 2013 and ground-truthing (131 sampling points) carried out in the winter of 2013-14. The classification was performed using Supervised Classification and Visual Revision method**

| **Land cover** | **Open waters** | **Mudflats** | **Wet meadows** | **Reed Marshes** | **User's accuracy** |
| --- | --- | --- | --- | --- | --- |
| **Open waters** | 13 | 0 | 0 | 0 | 1.00 |
| **Mudflats** | 2 | 14 | 6 | 1 | 0.61 |
| **Wet measdows** | 0 | 1 | 58 | 6 | 0.89 |
| **Reed Marshes** | 0 | 0 | 5 | 25 | 0.83 |
| **Producer's accuracy** | 0.87 | 0.93 | 0.84 | 0.78 |  |
| **Over all accuracy** | 83.96 |  |  |  |  |
| **Kappa** | 0.75 |  |  |  |  |

**Appendix 3 Autocorrelation (ACF) and partial autocorrelation functions (PACF) of the time series of monthly mean water level at Chenglingji Hydrological Station, suggesting strong autocorrelation among mean monthly water levels**


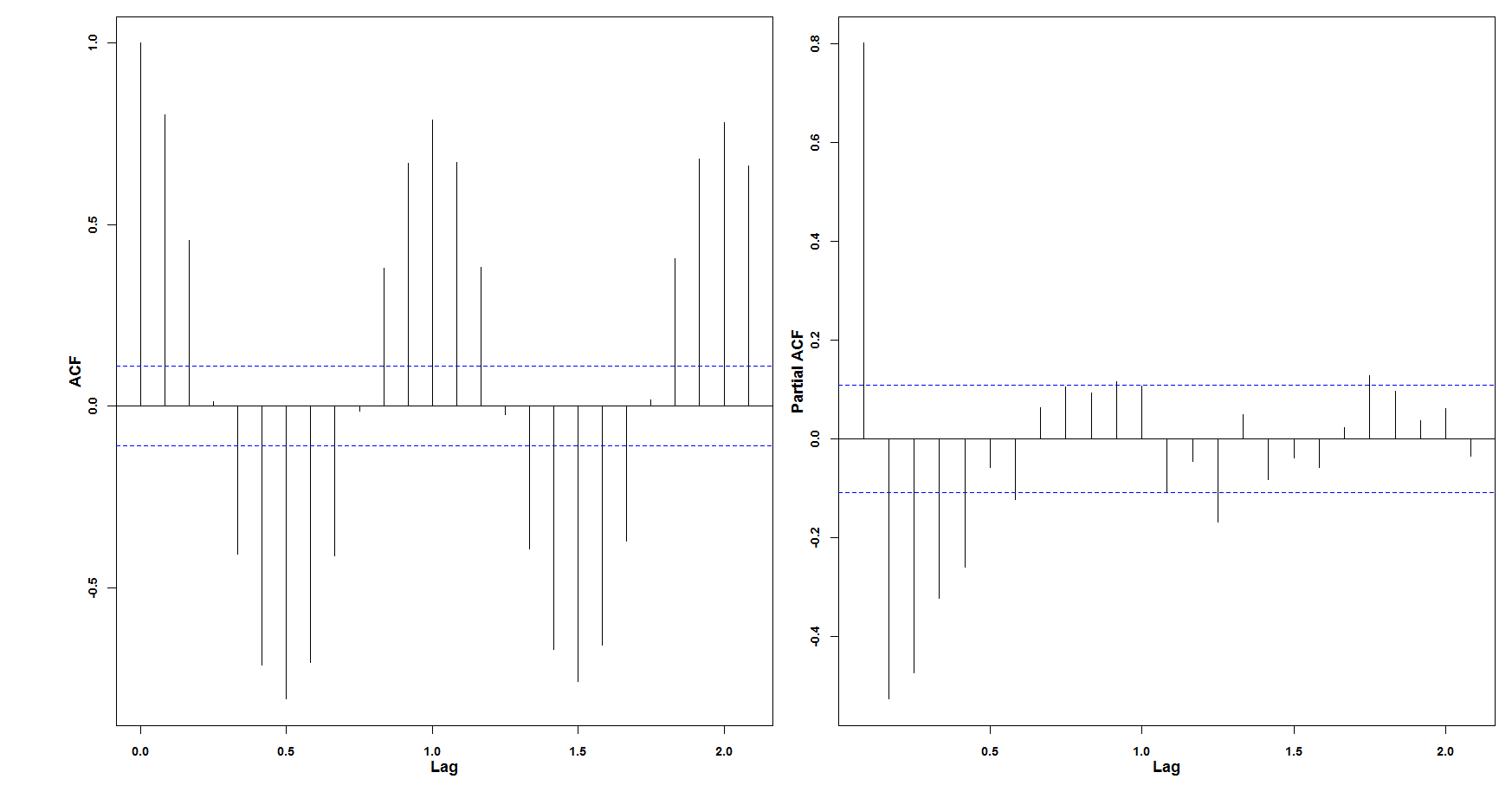

Supplement: Supplementary Information [file srep41761-s1.doc]
